# 

**Published:** 1824-02

**Authors:** 


### MONTHLY CATALOGUE OF MEDICAL BOOKS.

*** *It having been hinted to us that gentlemen, sending copies of their Works, prefer having their titles given at length, it is our intention, in future, to comply with this suggestion: but it is to be observed, that no books can be entered on this List except those sent to us for the purpose;—finding, in the lists hitherto transmitted, that the names of works have frequently been given as published, which have not appeared for weeks, or even months after.*

General Anatomy, applied to Physiology and the Practice of Medicine, by X. BICHAT. Translated from the last French Edition, by CONSTANT COFFYN. Revised and corrected by GEORGE CALVERT, Esq. Member of the Royal College of Surgeons. Part I. including the two first Volumes.—London, 1824. pp. 743.

An Essay on an improved Method of Cutting for Urinary Calculi, or the Posterior Operation of Lithotomy. By W. W. SLEIGH, Member of the Royal College of Surgeons, and Lecturer on Anatomy and Surgery in London, &c. &c.—London, 1824. pp. 104.

An Epitome of Chemistry, wherein the Principles of the Science are illustrated in One Hundred instructive and entertaining Experiments, capable of being performed without the Aid of any Apparatus except a few Wine-glasses, an Oil-flask, and a Crucible, and unattended with the least Danger. By the Rev. JOHN TOPHAM, M.A. (of St. John's College, Cambridge,) Head-master of Bromsgrove Grammar School, Worcestershire. Second Edition.—London. pp. 134.

A System of Anatomical Plates, accompanied with Descriptions, and Physiological and Pathological Observations. By JOHN LIZARS, F.R.S. Fellow of the R. C. of Surgeons, and Lecturer on Anatomy and Physiology.—Part I. the Bones. Part II. Blood-vessels and Nerves. Part III. ditto; with Plates in folio.—Edinburgh, 1823.

Memoire sur des Methodes et des Procedes nouveaux pour Pratiquer l'Amputation dans l'Articulation Scapulo-humerale. Par J. LISFRANC, &c.

Memoire sur des nouvelles Applications du Stethoscope de M. le Professeur Laennec. Par J. Lisfranc.—pp. 31.

An Essay on Apparitions, in which their Appearance is accounted for by Cases wholly independent of Preternatural Agency. By John Alderson, m.d. Senior Physician to the Hull General Infirmary; Consulting Physician to the Lying-in Charity; President of the Hull Literary and Philosophical Society; Honorary Member of the Yorkshire Philosophical Society; Corresponding Member of the Medico-Chirurgical Society of Edinburgh, &c. &c. New Edition, revised and corrected.—London, 1823. pp. 53.

